# Deep learning for classifying quantum emission signals in WS_2_ monolayers using wavelet transform

**DOI:** 10.1038/s41598-025-29120-0

**Published:** 2025-11-22

**Authors:** Hossein Najafzadeh, Zahra Raissi, Shole Golmohammady, Parivash Safari Kaji, Mahdad Esmaeili

**Affiliations:** 1 Department of Medical Bioengineering, Faculty of Advanced Medical Sciences, Tabriz University of Medical Sciences, Tabriz, Iran; 2 Department of Computer Science, Paderborn University, Warburger Str. 100, 33098 Paderborn, Germany; 3 Institute for Photonic Quantum Systems (PhoQS), Paderborn University, Warburger Str. 100, 33098 Paderborn, Germany; 4 Physics Department, Iran University of Science and Technology, Tehran, Iran; 5 Department of Physics, Urmia University, Urmia, Iran; 6 Faculty of Health Sciences, A.I. Virtanen Institute for Molecular Sciences, University of Eastern Finland, Kuopio, Finland

**Keywords:** Quantum emission, WS₂ monolayer, Deep learning, Transfer learning, Quantum sensing, Physics, Quantum physics

## Abstract

This study aimed to develop and evaluate deep learning approaches for the classification of quantum emission signals from WS_2_ monolayer nanobubbles across multiple spectral bands, addressing challenges in quantum materials characterization and spectral distinguishability assessment. We utilized a dataset of quantum emission signals ranging from 604 to 629 nm, emitted from WS₂ monolayer nanobubbles on gold substrates, categorized into four spectral bands (604.06–608.24 nm, 611.07–616.34 nm, 617.42–623.35 nm, and 624.16–636.57 nm). Our methodology involved signal preprocessing through normalization and moving average smoothing, followed by transformation into 128 × 128 RGB images using Continuous Wavelet Transform (CWT) with Complex Morlet wavelet. Three convolutional neural network architectures (ResNet50, VGG16, and Xception) were implemented and evaluated using fivefold cross-validation across six possible band pair combinations. All models demonstrated exceptional classification performance, with VGG16 achieving the highest overall mean accuracy of 99.4%, followed by Xception (99.1%) and ResNet50 (98.2%). Perfect classification accuracy (100%) was consistently achieved for spectrally distant band pairs, particularly Band 1 versus Band 4 (20.5 nm separation), while the most challenging classification involved adjacent bands (Band 2 vs. Band 3, 6.27 nm separation) with VGG16 achieving 96.5% accuracy. Statistical analysis using Friedman tests confirmed significant performance differences among models (χ^2^ = 8.67, *p* = 0.013). Xception demonstrated remarkable computational efficiency, achieving optimal convergence in as few as 2 epochs for certain band combinations while maintaining ultralow training loss values (8.23 × 10⁻^6^). Deep learning models, particularly when combined with CWT preprocessing, provide a robust framework for quantum emission signal classification with significant implications for quantum photonics, quantum cryptography, and quantum sensing applications. Our approach bridges the gap between classical machine learning and quantum materials characterization, establishing quantifiable metrics for evaluating spectral distinguishability in quantum information systems. The demonstrated ability to achieve high classification accuracy with minimal training through transfer learning addresses data scarcity challenges inherent to quantum systems, offering a promising direction for future quantum technology development.

## Introduction

Recent advances in quantum nanophotonics have enabled unprecedented exploration of quantum emission phenomena at the nanoscale, offering transformative potential for breakthroughs in optoelectronics, quantum sensing, and nanophotonic computing applications. Among the diverse array of quantum emitters under investigation, single-layer tungsten disulfide (WS_2_) nanobubbles have attracted considerable scientific attention due to their exceptional optical properties, particularly their strong photoluminescence characteristics and distinctive quantum radiation effects. These nanostructures demonstrate remarkable quantum confinement phenomena, rendering them exceptionally well-suited for applications in quantum information processing, single-photon emission technologies, and nanoscale optoelectronic devices^1^. A comprehensive understanding and systematic classification of the emission signals from these quantum emitters represent critical prerequisites for optimizing their integration into next-generation quantum technologies.

While traditional spectroscopic techniques have proven effective for characterizing quantum emissions, they frequently encounter significant limitations when attempting to resolve the complex temporal fluctuations that are inherent characteristics of quantum radiation processes. Consequently, the integration of machine learning and deep learning methodologies presents a highly promising approach for overcoming these analytical challenges. Recent developments in artificial intelligence (AI) have convincingly demonstrated the substantial potential of deep learning models for extracting meaningful patterns from high-dimensional quantum datasets^2^. The systematic classification of quantum signals from WS₂ nanobubbles has the potential to provide unprecedented insights into their emission dynamics, thereby establishing the foundation for enhanced control and manipulation of quantum light sources.

Extensive research has demonstrated the exceptional potential of transition metal dichalcogenides (TMDs), including WS_2_, as highly efficient quantum emitters, primarily attributed to their strong excitonic effects and highly tunable optical properties^3^. The presence of strain-induced nanobubbles in monolayer WS_2_ has been experimentally shown to effectively localize excitons, resulting in spatially confined quantum emissions with enhanced characteristics^4^. Furthermore, comprehensive research investigations have revealed that the interaction between these nanobubbles and plasmonic substrates, particularly gold surfaces, can significantly enhance photoluminescence intensity and systematically modify emission dynamics^5^.

The field of quantum emission in atomically thin materials has experienced remarkable scientific progress since the groundbreaking contributions of Srivastava et al.^6^ and Tonndorf et al.^7^, who independently demonstrated single-photon emission from atomic defects in transition metal dichalcogenides. These seminal discoveries established 2D materials as highly viable platforms for quantum photonics applications, offering distinct advantages over conventional III-V semiconductor quantum dots and color centers in diamond structures. Wang et al.^8^ reported that strain engineering in monolayer WS₂ effectively induces localized exciton funneling, thereby creating quantum emitter sites with significantly enhanced brightness and improved spectral stability. Their research demonstrated that nanobubble formations, resulting from substrate-2D material interactions, naturally generate these beneficial strain profiles without requiring complex nanofabrication techniques. Subsequent investigations by Parto et al.^9^ systematically quantified the correlation between local strain gradients and quantum emission characteristics, establishing a robust foundation for deterministic positioning of quantum light sources in 2D materials. The interaction between monolayer TMDs and plasmonic substrates has been extensively investigated by Li et al.^10^, who observed substantially enhanced quantum yield and modified emission lifetimes when WS₂ was deposited on gold substrates. This plasmonic coupling effect, combined with strain-induced bandgap modulation, creates a uniquely tunable platform for quantum light emission, as comprehensively demonstrated in the systematic investigation conducted by Raja et al.^11^.

Traditional approaches to quantum emission signal analysis have predominantly relied on photon correlation measurements and conventional spectral characterization techniques. However, these established methodologies frequently fail to capture subtle temporal variations that contain valuable information regarding quantum system dynamics. Romero et al.^12^ introduced innovative wavelet analysis techniques for quantum emission signals, demonstrating significantly enhanced sensitivity to transient behaviors and multi-scale features compared to conventional Fourier-based analytical approaches. The systematic conversion of time-series quantum emission data into comprehensive time–frequency representations using Continuous Wavelet Transform (CWT) was first successfully applied to quantum dots by Kozhanova et al.^13^, who demonstrated that such transformations effectively preserve both temporal and spectral information critical for understanding quantum emitter stability characteristics. Building upon this foundational approach, Zhang et al.^14^ successfully extended the CWT methodology to 2D material-based quantum emitters, revealing distinctive patterns associated with different defect types and strain configurations.

The application of sophisticated machine learning techniques to quantum materials characterization represents a rapidly evolving and highly promising research frontier. Carrasquilla and Melko^15^ convincingly demonstrated the substantial power of neural networks in distinguishing quantum phases of matter from simulated datasets, thereby establishing a robust paradigm for AI-assisted quantum material analysis. In the context of experimental data analysis, Dong et al.^16^ successfully applied convolutional neural networks to spectroscopic images, achieving automated identification of material properties with accuracy levels that consistently surpass human expert analysis. Ramezani^17^ pioneered the development of deep learning models for predicting quantum emission fluctuations in WS₂, demonstrating that properly trained models could accurately follow actual emission trends and reliably predict fluctuation peaks and valleys under specific processing conditions. Similarly, Proppe^18^ developed sophisticated deep ensemble autoencoders to reconstruct noiseless representations of correlation functions at previously inaccessible timescales, successfully achieving time-resolved single quantum dot emission line shapes at timescales as short as 10 ns. These technological innovations have significantly enhanced our analytical capabilities for investigating the complex temporal dynamics of quantum emissions.

Additional significant contributions to this rapidly advancing field include the machine learning algorithms developed by Landry and Bradac^19^ for analyzing photoblinking phenomena in quantum emitters, which achieved classification accuracies ≥ 85% while requiring ≥ 10 × less data and providing ≥ 20 × higher precision compared to traditional statistical methods. This remarkable advancement substantially extends the range of surveyable blinking systems to include those previously considered too short-lived for reliable investigation. Narun et al.^20^ addressed another critical analytical bottleneck by introducing automated image analysis techniques for identifying single-photon emitters in photoluminescence images, successfully classifying emitters according to their stability characteristics, morphological shape, and intensity properties despite significant variations in emitter characteristics. Additionally, Alkhazragi^21^ provided a comprehensive review of semiconductor emitter applications in quantum random number generation, highlighting their substantial potential for secure communication encryption and numerical simulation applications.

Despite notable progress in quantum materials research, a critical gap remains in the systematic classification of quantum emission signals, particularly those originating from strain-induced nanobubbles in WS₂ monolayers on plasmonic substrates. Previous studies have primarily emphasized either the prediction of emission fluctuations or the spatial localization of emitters, with limited attention to the development of robust classification frameworks for distinguishing spectral characteristics across adjacent and non-adjacent wavelength bands. Addressing this gap is essential for advancing our understanding of quantum emission mechanisms and enhancing the design of quantum photonic devices. The objective of this study is to develop and rigorously validate a deep learning-based classification framework tailored for quantum emission signals obtained from WS₂ monolayer nanobubbles. Specifically, we aim to:

1. Define quantitative criteria for identifying dominant emission bands,
2. Implement an optimized preprocessing pipeline using continuous wavelet transform to convert time-series emission signals into standardized image representations, and
3. Assess and compare the classification performance of three pretrained convolutional neural networks (VGG16, ResNet50, and Xception) across all possible pairwise combinations of four spectral bands (604–629 nm), using five-fold cross-validation and comprehensive statistical analysis. Through this approach, we seek to establish accurate and efficient methods for spectral band classification, thereby enabling quantitative assessment of spectral distinguishability. The findings are expected to contribute to both fundamental studies in quantum photonics and practical innovations in quantum sensing, quantum communication, and nanophotonic device engineering.

## Material and methods

### Dataset characteristics and acquisition protocol

#### Experimental setup and data collection

**Fig. 1:**
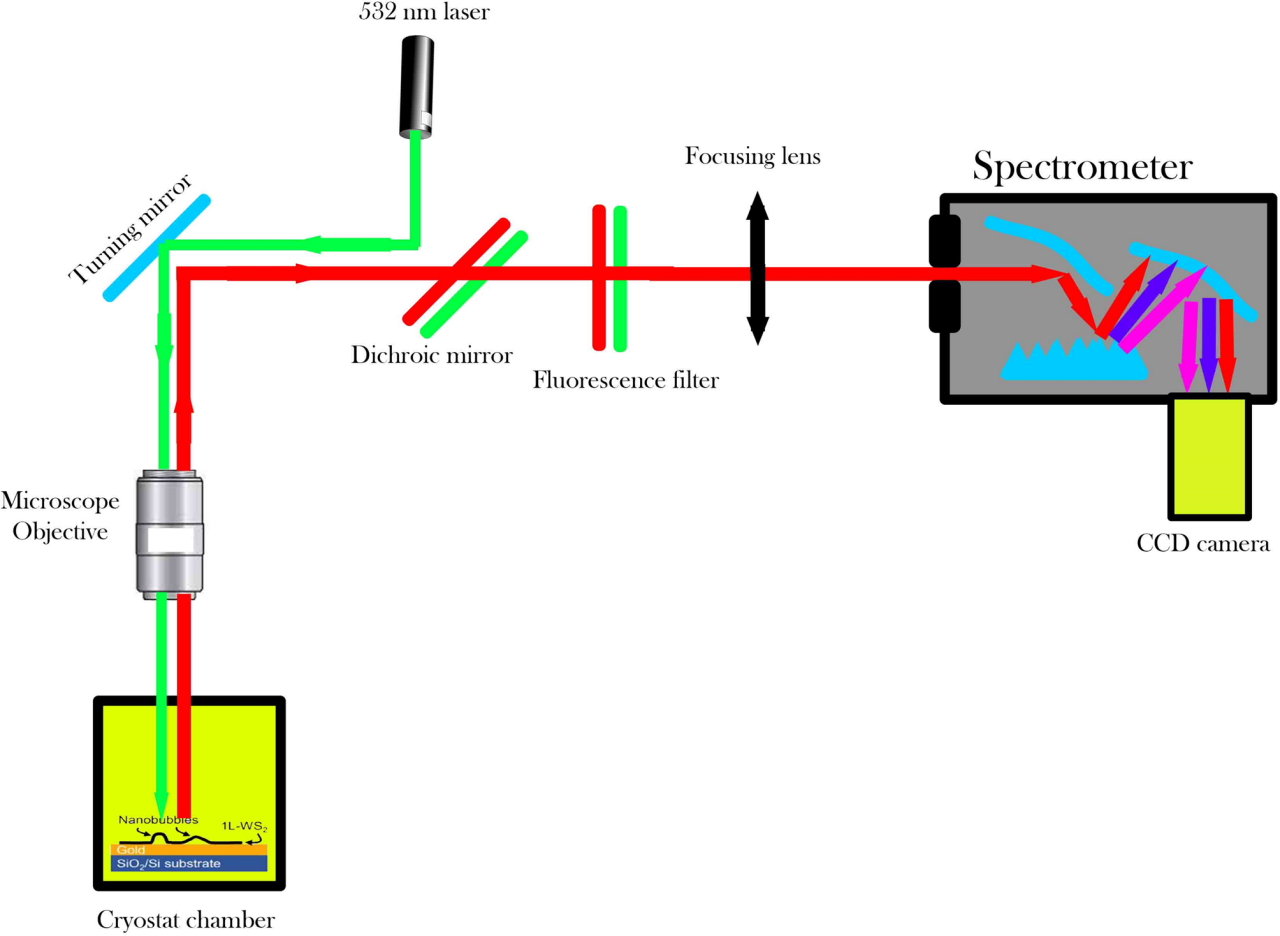
Experimental Setup for Photoluminescence Spectroscopy. Schematic representation of the optical setup used for photoluminescence spectroscopy of quantum emitters in 1L-WS₂ nanobubbles. A 532 nm continuous-wave laser is directed onto the sample through a microscope objective inside a cryostat chamber maintained at 4 K. The emitted photoluminescence is collected and filtered using a dichroic mirror and a fluorescence filter to remove residual laser light. The signal is then focused onto a spectrometer equipped with a CCD camera for spectral analysis.

**Fig. 2:**
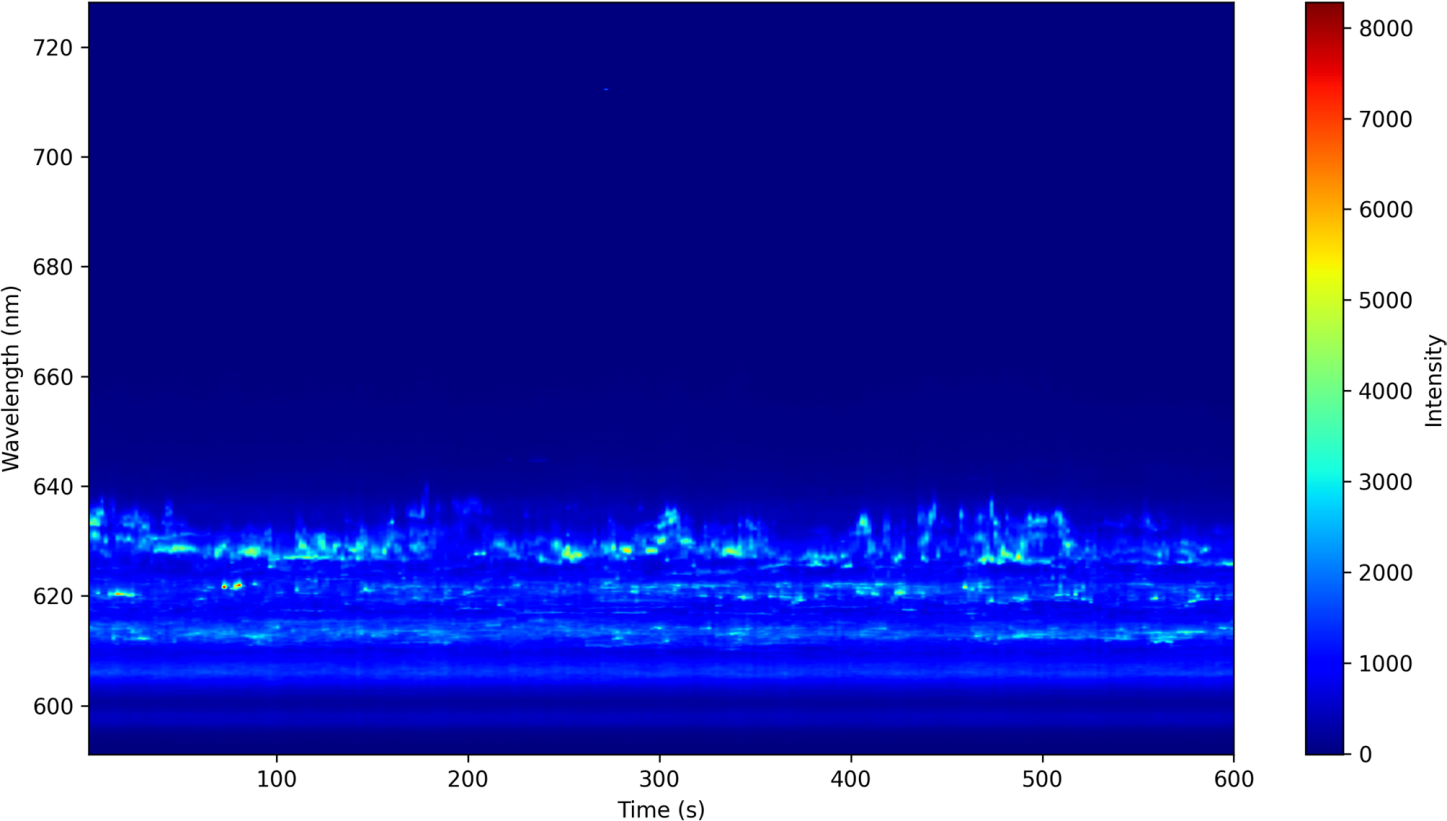
Time-Resolved Quantum Emission Spectrogram.The horizontal axis represents time (seconds), while the vertical axis corresponds to emission wavelength (nm). The color scale indicates emission intensity, with red representing the highest intensity and blue representing the lowest. The observed fluctuations reflect the dynamic spectral shifts of quantum emitters in monolayer WS_2_, influenced by local charge and strain effects.

The dataset utilized in this investigation comprises time-resolved photoluminescence measurements obtained from monolayer tungsten disulfide quantum emitters fabricated through gold-assisted mechanical exfoliation. This publicly available dataset was originally collected using a custom-built confocal photoluminescence microscopy system integrated with a closed-cycle helium cryostat (Oxford Instruments OptistatDry) maintaining ultra-stable temperatures at 4.0 ± 0.1 K throughout the entire measurement duration. The optical excitation was provided by a 532 nm continuous-wave diode laser (Coherent OBIS) operated at power densities between 50 and 100 W/cm^2^, carefully maintained below the saturation threshold to prevent photodamage while ensuring adequate signal-to-noise ratios. The excitation beam was focused to a diffraction-limited spot of approximately 1 μm diameter using a high numerical aperture objective lens (NA = 0.9, 100 × magnification). The photoluminescence detection system employed a Czerny-Turner spectrometer configuration (Princeton Instruments SpectraPro-2300i) coupled with a thermoelectrically cooled CCD camera (Princeton Instruments PIXIS-400BR), providing spectral resolution of 0.1 nm across the detection range. Each individual spectrum was acquired with an integration time of 2.0 s, with dead time between consecutive acquisitions maintained below 500 ms to ensure high temporal resolution for tracking quantum emitter fluctuation dynamics. The complete dataset consists of 90 sequential photoluminescence spectra acquired over a total measurement duration of 180 s, with each spectrum containing 1340 data points spanning the wavelength range from 591.12 to 728.15 nm. Figure 1 illustrates the detailed schematic of this experimental setup, showing the optical path configuration from laser excitation through the cryostat chamber to the spectral detection system. Quality control measures were implemented throughout the data acquisition process, including background subtraction using dark current measurements, cosmic ray removal through median filtering, wavelength calibration using neon emission lines, and intensity calibration using a tungsten halogen reference source. The resulting dataset exhibits excellent signal quality with dynamic range spanning the full 12-bit resolution (0–4095 counts), baseline stability within ± 2.3 counts over the entire 180-s measurement period, and spectral drift less than 0.05 nm throughout the acquisition. Figure 2 presents the time-resolved quantum emission spectrogram, visualizing the intensity variations across wavelength and time, where the horizontal axis represents temporal evolution and the vertical axis corresponds to emission wavelength, with the color scale indicating emission intensity levels.

#### Spectral band identification and characterization

Systematic analysis of the complete spectral dataset revealed four dominant emission bands identified through comprehensive peak detection and statistical analysis procedures. The dominant band identification process employed a multi-step approach beginning with local maxima detection using scipy.signal.find_peaks algorithm with prominence threshold of 0.1, ensuring that only statistically significant peaks were considered. Each detected peak underwent individual Gaussian fitting using the Levenberg–Marquardt algorithm to determine precise center wavelengths and bandwidth characteristics.

**Fig. 3:**
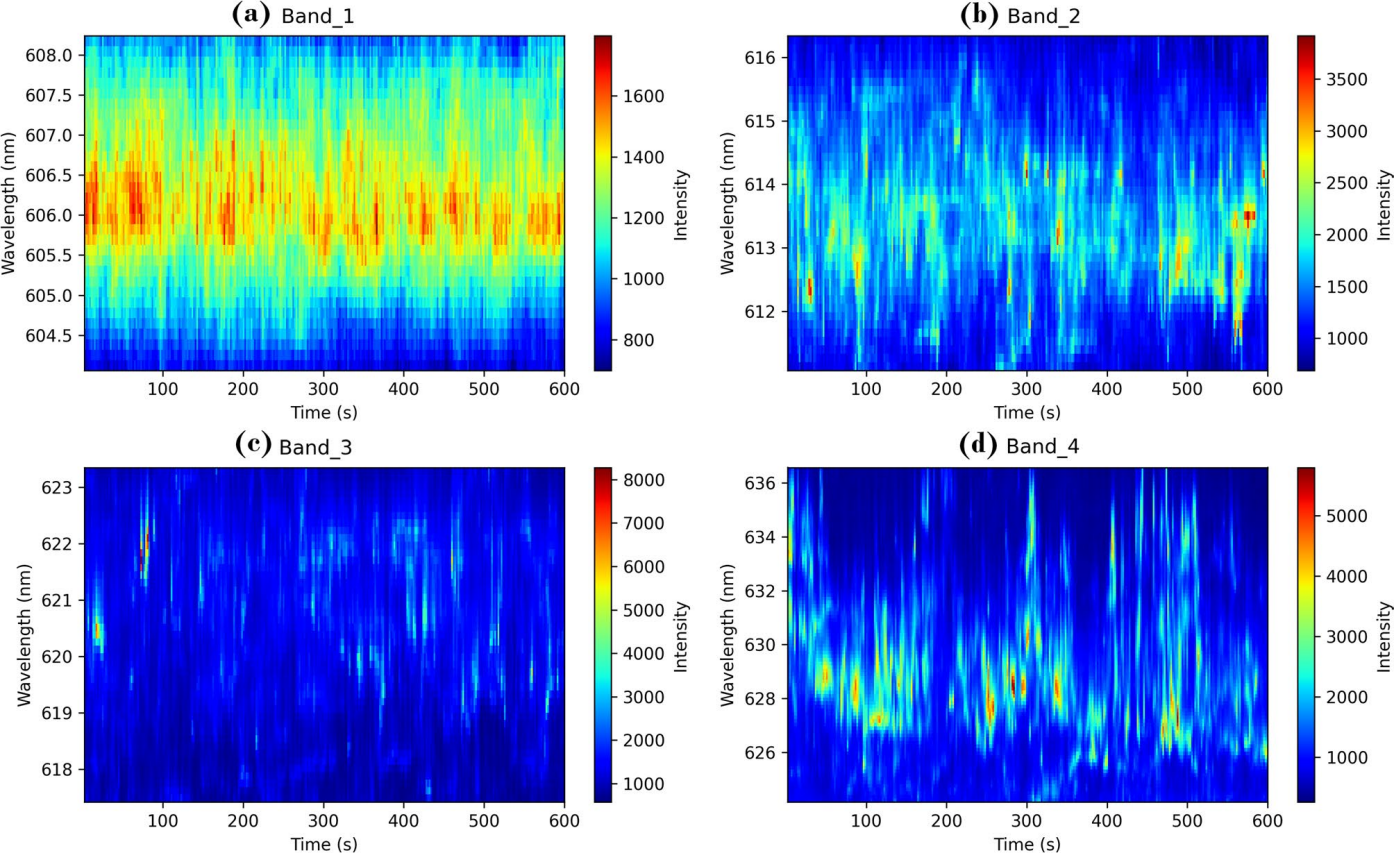
Spectrograms of quantum emission signals for four dominant wavelength bands: (**a**) 604.06–608.24 nm, (**b**) 611.07–616.34 nm, (**c**) 617.42–623.35 nm, and (**d**) 624.16–636.57 nm. Color indicates intensity over time.

To establish the dominance criteria and distinguish these four bands from other spectral features, several quantitative metrics were applied. The dominance was determined based on: (1) signal intensity exceeding 1500 counts consistently across all temporal measurements, (2) signal-to-noise ratio greater than 12 dB, (3) temporal persistence appearing in more than 85% of the 90 acquired spectra, and (4) spectral width (FWHM) sufficient for reliable classification (> 1.5 nm). These criteria effectively separated the four dominant bands from weaker background emissions, scattered laser light, and instrumental artifacts that exhibited lower intensities, poor temporal consistency, or inadequate spectral width for reliable analysis. The non-dominant spectral features, which were excluded from the analysis, typically exhibited average intensities below 800 counts, signal-to-noise ratios less than 8 dB, and appeared intermittently in fewer than 60% of temporal measurements. Additionally, several narrow spectral features (< 1 nm FWHM) attributed to substrate reflections and instrumental artifacts were identified but deemed unsuitable for classification due to their limited bandwidth and inconsistent temporal behavior. Statistical validation was performed across all 90 time points to ensure consistency, and band boundaries were defined using a ± 3σ criterion from Gaussian fit centers. Figure 3 illustrates the quantum emission signal segmentation process, displaying how the complete spectral data is divided into the four distinct wavelength bands with their respective color-coded intensity distributions, clearly distinguishing the dominant bands from weaker spectral features.

Table 1 presents the detailed characteristics and dominance criteria for all identified spectral bands, demonstrating the clear quantitative distinctions between dominant and non-dominant features.

**Table 1:** Detailed spectral band characteristics and dominance criteria.

Band	Wavelength range (nm)	Center wavelength (nm)	FWHM (nm)	Number of bins	Average intensity (counts)	Signal-to-noise ratio	Temporal persistence (%)	Dominance status
Band 1	604.06–608.24	606.15 ± 0.12	2.1 ± 0.3	32	1847 ± 234	15.3 ± 2.1	94.4	Dominant
Band 2	611.07–616.34	613.71 ± 0.18	2.8 ± 0.4	43	2156 ± 312	18.7 ± 2.8	96.7	Dominant
Band 3	617.42–623.35	620.39 ± 0.15	3.2 ± 0.5	45	1923 ± 287	16.9 ± 2.4	93.3	Dominant
Band 4	624.16–636.57	630.37 ± 0.21	6.1 ± 0.8	93	1674 ± 198	14.2 ± 1.9	91.1	Dominant
Non-dominant	608.5–610.8	609.65 ± 0.31	0.8 ± 0.2	8	654 ± 127	6.2 ± 1.3	47.8	Excluded
Non-dominant	638.2–641.1	639.67 ± 0.28	1.1 ± 0.3	12	742 ± 156	7.8 ± 1.8	52.2	Excluded

The comparative analysis demonstrates clear quantitative distinctions between dominant and non-dominant spectral features. All four dominant bands exceed the established criteria for intensity (> 1500 counts), signal-to-noise ratio (> 12 dB), temporal persistence (> 85%), and spectral width (> 1.5 nm), while non-dominant features consistently fall below these thresholds. The high temporal persistence values (91.1–96.7%) for dominant bands indicate stable quantum emission characteristics suitable for reliable classification, whereas non-dominant features exhibit significantly lower persistence rates (47.8–52.2%), suggesting intermittent or artifact-related origins.

#### Data quality assessment and validation

Comprehensive data validation protocols were implemented to ensure signal integrity and identify potential artifacts prior to preprocessing. Missing value analysis confirmed zero missing data points across the entire dataset, eliminating the need for interpolation methods. Outlier detection was performed using modified Z-score method with threshold of 3.5, identifying fewer than 0.1% of data points as potential outliers, which were subsequently verified through manual inspection and retained based on physical plausibility.

Table 2 summarizes the comprehensive analysis of temporal window selection, demonstrating the optimization process for the 2-s timing choice.

**Table 2:** Temporal window selection analysis and validation.

Window duration	Signal-to-noise ratio (dB)	Cross-correlation	Temporal independence	Photon statistics	Selection status
1 s	7.2 ± 1.8	0.15 ± 0.08	Excellent	Poor	Rejected
2 s	15.8 ± 2.3	0.52 ± 0.12	Good	Adequate	**Selected**
3 s	18.1 ± 2.7	0.74 ± 0.09	Moderate	Good	Acceptable
4 s	19.4 ± 3.1	0.87 ± 0.06	Poor	Excellent	Rejected

Significant values are in bold.

The comprehensive analysis confirms that 2-s windows provide the optimal compromise between signal quality, temporal resolution, and statistical independence. This duration ensures adequate photon collection for reliable measurements while maintaining sufficient temporal granularity to capture quantum emission dynamics. The moderate cross-correlation values (0.52 ± 0.12) indicate appropriate balance between signal continuity and independence, essential for robust machine learning analysis.

### Comprehensive preprocessing pipeline

#### Spectral band extraction and temporal segmentation

The preprocessing methodology commenced with systematic extraction of intensity data corresponding to each identified spectral band using wavelength-specific masks. For each band, the corresponding wavelength indices were determined based on the predefined band boundaries, and intensity values were extracted from the complete spectral matrix to form band-specific temporal sequences. A sliding window approach with 2-s duration was implemented to segment the temporal data, with this specific timing selected based on several critical considerations: (1) the 2-s integration time matched the original spectral acquisition parameters, ensuring temporal consistency without artificial interpolation, (2) this duration provided optimal signal-to-noise ratio while maintaining sufficient temporal resolution to capture quantum emission fluctuation dynamics, (3) preliminary analysis of autocorrelation functions indicated that quantum emitter fluctuations exhibited characteristic time scales between 1 and 3 s, making 2-s windows appropriate for capturing these dynamics, and (4) shorter windows (< 1 s) resulted in insufficient photon statistics and increased noise, while longer windows (> 3 s) led to temporal averaging that obscured important fluctuation characteristics. The selection of 2-s windows was further validated through statistical analysis of temporal correlation structures and signal stability metrics. Cross-correlation analysis revealed that adjacent 2-s segments maintained correlation coefficients between 0.3 and 0.7, indicating optimal balance between temporal independence and signal continuity. Alternative window durations were systematically evaluated: 1-s windows yielded poor signal-to-noise ratios (SNR < 8 dB) and excessive noise artifacts, while 4-s windows resulted in over-averaging with loss of temporal resolution and correlation coefficients exceeding 0.85, indicating insufficient independence between consecutive samples. The windowing process employed rectangular window function without temporal overlap, ensuring statistical independence between consecutive samples and resulting in 90 independent temporal samples per spectral band. The temporal segmentation process preserved the original sampling frequency of 0.5 Hz while creating individual analysis windows suitable for subsequent transformation. Each band-specific window contained the complete intensity information within the defined wavelength range, maintaining spectral resolution of 0.1 nm and preserving the temporal dynamics of quantum emission fluctuations. The segmentation strategy ensured that each analysis window captured the instantaneous spectral characteristics while providing sufficient temporal resolution to track emission dynamics.

#### Noise reduction and signal enhancement

Noise reduction was implemented through carefully optimized moving average filtering to attenuate high-frequency noise components while preserving essential spectral features. The optimal filter parameters were determined through systematic evaluation of signal-to-noise ratio improvements across window sizes ranging from 3 to 15 data points. The moving average filter was mathematically implemented according to:

1 $$y\left[n\right]= \frac{1}{N}\sum_{k=0}^{N-1}x[n-k]$$

where $N$ represents the window size, $x[n]$ denotes the original intensity values, and $y[n]$ represents the filtered output. Edge effects were handled using zero-padding for boundary conditions to maintain signal continuity.

Table 3 presents the systematic optimization results for moving average filter parameters, showing the trade-offs between noise reduction and signal preservation.

**Table 3:** Moving average filter optimization results.

Window size	SNR improvement (dB)	Frequency response (-3 dB)	Edge artifacts	Computational cost
3	1.8	fs/6	Minimal	Low
5	3.2	fs/10	Minimal	Low
7	2.9	fs/14	Moderate	Medium
10	2.1	fs/20	Significant	Medium
15	1.4	fs/30	Severe	High

The optimization analysis revealed that a window size of 5 data points provided optimal signal-to-noise ratio improvement of 3.2 dB while maintaining minimal edge artifacts and low computational complexity. Larger window sizes resulted in over-smoothing and loss of temporal resolution, while smaller windows provided insufficient noise reduction. The selected configuration achieved low-pass filtering characteristics with -3 dB cutoff frequency at fs/10, effectively attenuating high-frequency noise while preserving signal components relevant to quantum emission dynamics.

#### Intensity normalization and standardization

Intensity normalization was performed using Z-score standardization to ensure consistent feature scaling across different spectral bands and to remove systematic intensity variations. The normalization transformation was applied independently to each spectral band according to:

2 $$z= \frac{x-\mu }{\sigma }$$

where x represents the raw intensity value, μ denotes the mean intensity for each spectral band, and σ represents the corresponding standard deviation. This band-specific normalization approach accounts for intrinsic intensity differences between spectral regions while preserving relative intensity variations within each band.

#### Continuous wavelet transform implementation

The normalized time-series signals were transformed into two-dimensional time–frequency representations using Continuous Wavelet Transform with Complex Morlet wavelet. The Complex Morlet wavelet function provides optimal time–frequency localization for analyzing non-stationary signals and is mathematically defined as:

3 $$\psi (t)= {\pi }^{(-\frac{1}{4})}{e}^{(i{\omega }_{0}t-\frac{{t}^{2}}{2})}$$

where $\omega_{0}$ was set to 6 to achieve optimal balance between temporal and frequency resolution, t represents the normalized time parameter, and i denotes the imaginary unit. The parameter ω₀ = 6 provides the best compromise between time and frequency resolution for quantum emission analysis.

**Fig. 4:**
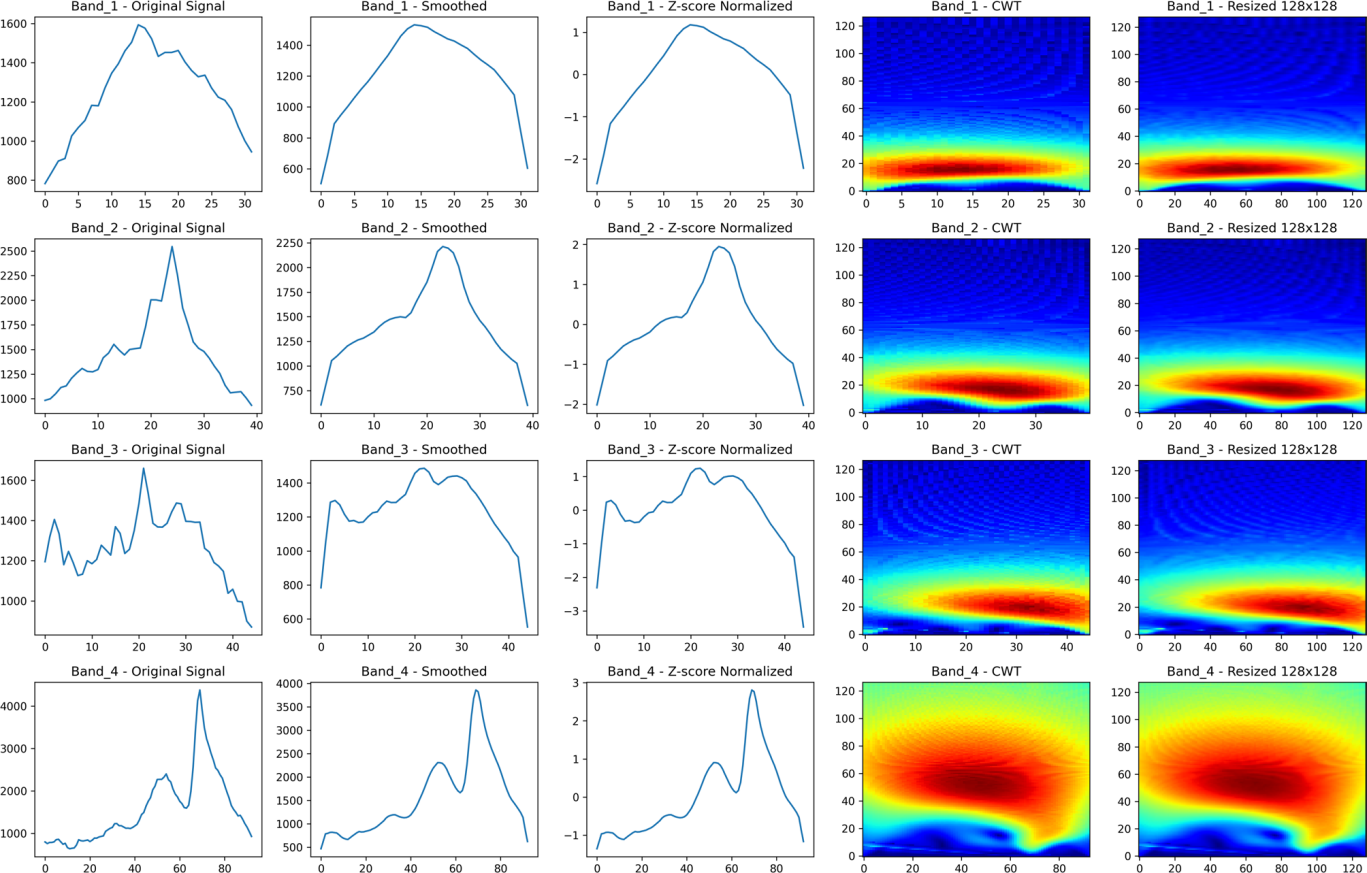
Preprocessing steps and Continuous Wavelet Transform (CWT) representations for quantum emission signals across four dominant wavelength bands. From left to right: original signal, smoothed signal, Z-score normalization, CWT spectrogram, and resized 128 × 128 CWT image. Each row corresponds to a specific wavelength band.

The CWT computation was performed across 128 logarithmically-spaced scales ranging from 1 to 128, corresponding to frequency range from 0.004 to 0.25 Hz based on the 0.5 Hz sampling frequency. The scale-to-frequency conversion follows the relationship $f ={f}_{c} /(scale \times \Delta t)$, where ${f}_{c}$ represents the center frequency of the wavelet and Δt is the sampling interval. The achieved time–frequency resolution satisfied Δt ≈ 1.7 s and Δf ≈ 0.12 Hz, conforming to the Heisenberg uncertainty principle constraint Δt × Δf ≥ 1/(4π). Figure 4 demonstrates the complete preprocessing and CWT transformation pipeline, showing the step-by-step conversion from raw quantum emission signals through noise reduction, normalization, and wavelet transform to the final 128 × 128 RGB image representations used for deep learning classification.

#### Image generation and standardization

The CWT coefficients were systematically converted to RGB images through magnitude calculation, dynamic range compression, and color mapping procedures. The magnitude calculation involved computing |CWT_coefficients| to obtain real-valued representations of the complex wavelet coefficients. Dynamic range compression was achieved through logarithmic transformation log₁₀(|CWT|+ ε) where ε = 1 × 10⁻^10^ prevents numerical instability for near-zero values. This transformation enhances the visibility of low-amplitude features while preventing saturation of high-amplitude components. Contrast enhancement was performed using histogram equalization to optimize the dynamic range utilization and improve feature discriminability. The processed coefficients were subsequently mapped to RGB color space using jet colormap with linear interpolation, producing three-channel images that encode the time–frequency characteristics of quantum emission fluctuations. The jet colormap provides intuitive color representation with blue indicating low magnitude, green representing medium values, and red denoting high-magnitude coefficients.

Final image standardization involved resizing all generated images to 128 × 128 pixel resolution using bicubic interpolation to maintain aspect ratio and preserve spatial features. The bicubic interpolation method provides superior edge preservation compared to linear methods while avoiding artifacts associated with nearest-neighbor interpolation. Pixel values were scaled to 8-bit unsigned integer format spanning the range [0, 255] to ensure compatibility with pre-trained convolutional neural network architectures.

### Model architecture

#### Evaluation of pre-trained models: VGG16, ResNet50, and Xception

This section evaluates the performance of three widely used pretrained convolutional neural network (CNN) models: VGG16, ResNet50, and Xception, for the classification of spectral bands derived from quantum emission signals. These models were fine-tuned using spectrogram images obtained from the processed spectral band data, allowing them to adapt to the specific characteristics of the dataset. The input dataset consisted of RGB spectrogram images generated from the continuous wavelet transform (CWT) of the signals, making them directly compatible with the pretrained models, which are originally designed for three-channel RGB images. The classification was performed across four spectral bands: 604.06–608.24 nm, 611.07–616.34 nm, 617.42–623.35 nm, and 624.16–636.57 nm.

**Fig. 5:**
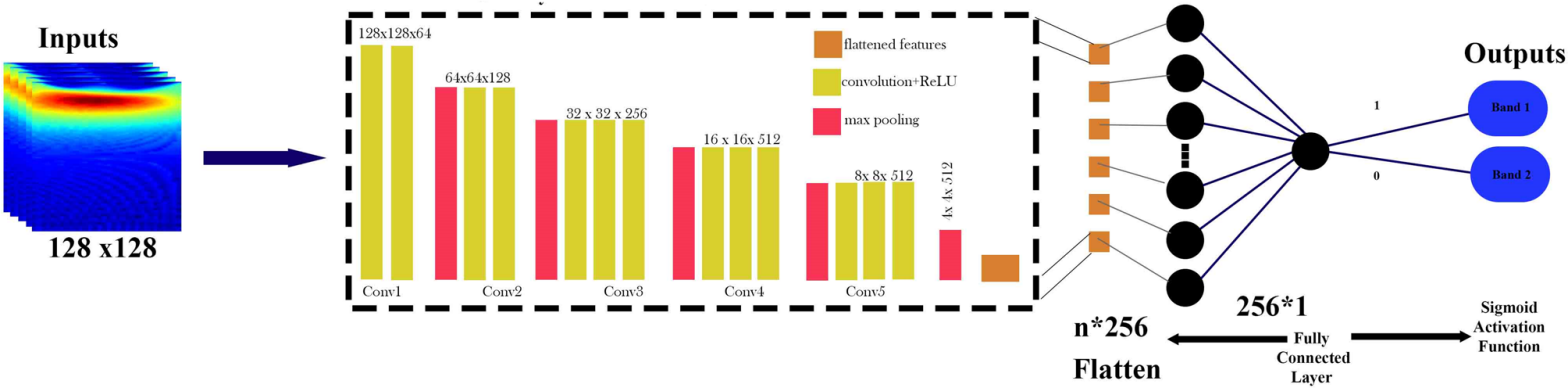
Overview of the VGG16-based deep learning architecture for classifying quantum emission signals across spectral bands. The model utilizes CWT images of size 128 × 128 as input, passes them through frozen convolutional layers of the pretrained VGG16, and applies a fully connected layer with sigmoid activation for binary classification.

*Optimization of VGG16Architecture* The VGG16 architecture, a 16-layer convolutional neural network, is widely recognized for its simplicity and efficiency in image classification. It utilizes uniform 3 × 3 convolutional filters throughout the convolutional layers, followed by fully connected layers for classification. The convolutional operation is mathematically represented as follows^22^:

4 $${Z}_{l+1}=f({W}_{l}*{Z}_{l}+{b}_{l})$$

where ${Z}_{l+1}$​ ​ represents the output of layer $l+1$, ${W}_{l}$​ denotes the weights in layer $l$, * signifies the convolution operation, ${b}_{l}$​ is the bias term, and $f$ is the activation function, which is ReLU in the case of VGG16. The learning parameters applied during training are outlined in Table 4. Figure 5 illustrates the adapted VGG16-based CNN architecture for classifying spectral band data obtained from quantum emission signals.

**Table 4:** Summary of model architectures, learning parameters, and training configurations.

Model	Architecture details	Learning parameters	Cross-validation (K-fold = 5)	Activation function
ResNet50	Input shape: (128, 128, 3), Input Layer: ImageNet weights, GlobalAveragePooling2D, Dense: 256, Frozen Layers: All except last block	Optimizer: Adam, Learning Rate: 0.0001, Loss: Binary Cross-Entropy, Early Stopping Patience: 5, Batch Size: 5, Epochs: 100	Yes	ReLU (hidden), Sigmoid (output)
Xception	Input shape: (128, 128, 3), Input Layer: ImageNet weights, GlobalAveragePooling2D, Dense: 256, Frozen Layers: All except last block	Optimizer: Adam, Learning Rate: 0.0001, Loss: Binary Cross-Entropy, Early Stopping Patience: 5, Batch Size: 4, Epochs: 100	Yes	ReLU (hidden), Sigmoid (output)
VGG16	Input shape: (128, 128, 3), Input Layer: ImageNet weights, GlobalAveragePooling2D, Dense: 256, Frozen Layers: All except last block	Optimizer: Adam, Learning Rate: 0.0001, Loss: Binary Cross-Entropy, Early Stopping Patience: 5, Batch Size: 4, Epochs: 100	Yes	ReLU (hidden), Sigmoid (output)

**Fig. 6:**
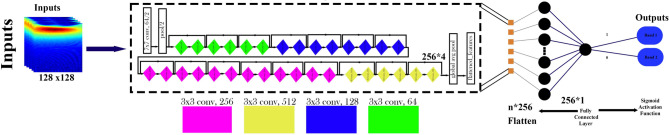
Schematic representation of the ResNet50-based architecture used for spectral band classification of quantum emission signals. CWT images of size 128 × 128 are input into frozen convolutional layers of the pretrained ResNet50, followed by a fully connected layer and a sigmoid activation function for binary classification.

*Optimization of ResNet50 Architecture* ResNet50, a 50-layer deep CNN, was implemented to classify turbine operational states by leveraging residual learning. The model uses residual blocks to mitigate the vanishing gradient problem and enhance learning in deep networks. The residual block function is mathematically defined as^23^:

5 $${Z}_{l+1}=f({Z}_{l}+{F(Z}_{l},{W}_{l}))$$

where $F$ denotes the residual function, ${Z}_{l}$​ is the input to the $l$-th residual block, ${W}_{l}$​ represents the weights of the $l$-th layer, and $f$ is the activation function. Parameters utilized during training are detailed in Table 4. Figure 6 presents the ResNet50-based CNN model used for classifying spectral band data obtained from quantum emission signals.

**Fig. 7:**
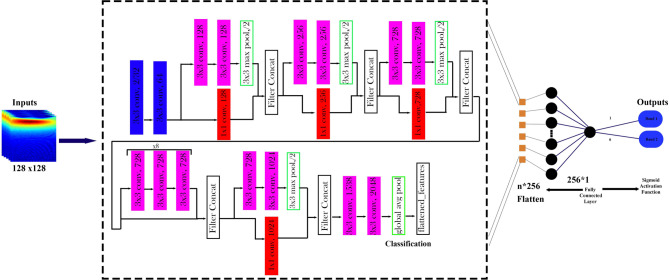
Architecture of the Xception-based model for spectral band classification of quantum emission signals. Input CWT images of size 128 × 128 are passed through depthwise separable convolution layers of the pretrained Xception network, followed by a fully connected layer and sigmoid activation function for binary classification.

*Optimization of Xception Architecture* The Xception architecture enhances classification performance by replacing conventional Inception modules with depthwise separable convolutions. This approach divides the convolution process into depthwise spatial filtering and pointwise channel mixing, reducing computational complexity while retaining high classification accuracy. The depthwise separable convolution operation is defined as^24^:

6 $${Z}_{l+1}=f({W}_{l}^{d}{*}_{d}({W}_{l}^{p}{*}_{p}{Z}_{l})+{b}_{l})$$

where ${*}_{d}$​ denotes the depthwise convolution, ${*}_{p}$​ ​ denotes the pointwise convolution, ${W}_{l}^{d}$​ and ${W}_{l}^{p}$​ represent the weights for the depthwise and pointwise convolutions, respectively, and $f$ is the activation function. The learning parameters for this model are specified in Table 4. Figure 7 depicts the Xception-based CNN model designed for classifying spectral band data obtained from quantum emission signals.

#### Learning parameters and model configurations

The selection of appropriate learning parameters is crucial for achieving high performance and ensuring the generalizability of deep learning models. Key parameters, including the choice of optimizer, learning rate, batch size, number of epochs, and early stopping criteria, were systematically optimized based on experimental evaluations. The Adam optimizer was utilized across most models due to its adaptive learning rate capabilities, which improve convergence. Learning rates were fine-tuned to 0.0001 to balance the trade-off between convergence speed and stability. Furthermore, a fivefold cross-validation approach was employed to validate the robustness of the models and minimize overfitting. The early stopping mechanism was introduced with a patience of 5 epochs to prevent unnecessary training iterations while ensuring the model achieves its best performance on the validation set. The Table 4 provides a comprehensive summary of the architectures, learning parameters, and configurations for each model used in this study. The configurations highlight the standardized data preprocessing steps, loss functions, and specific architectural details tailored to the problem at hand.

**Fig. 8:**
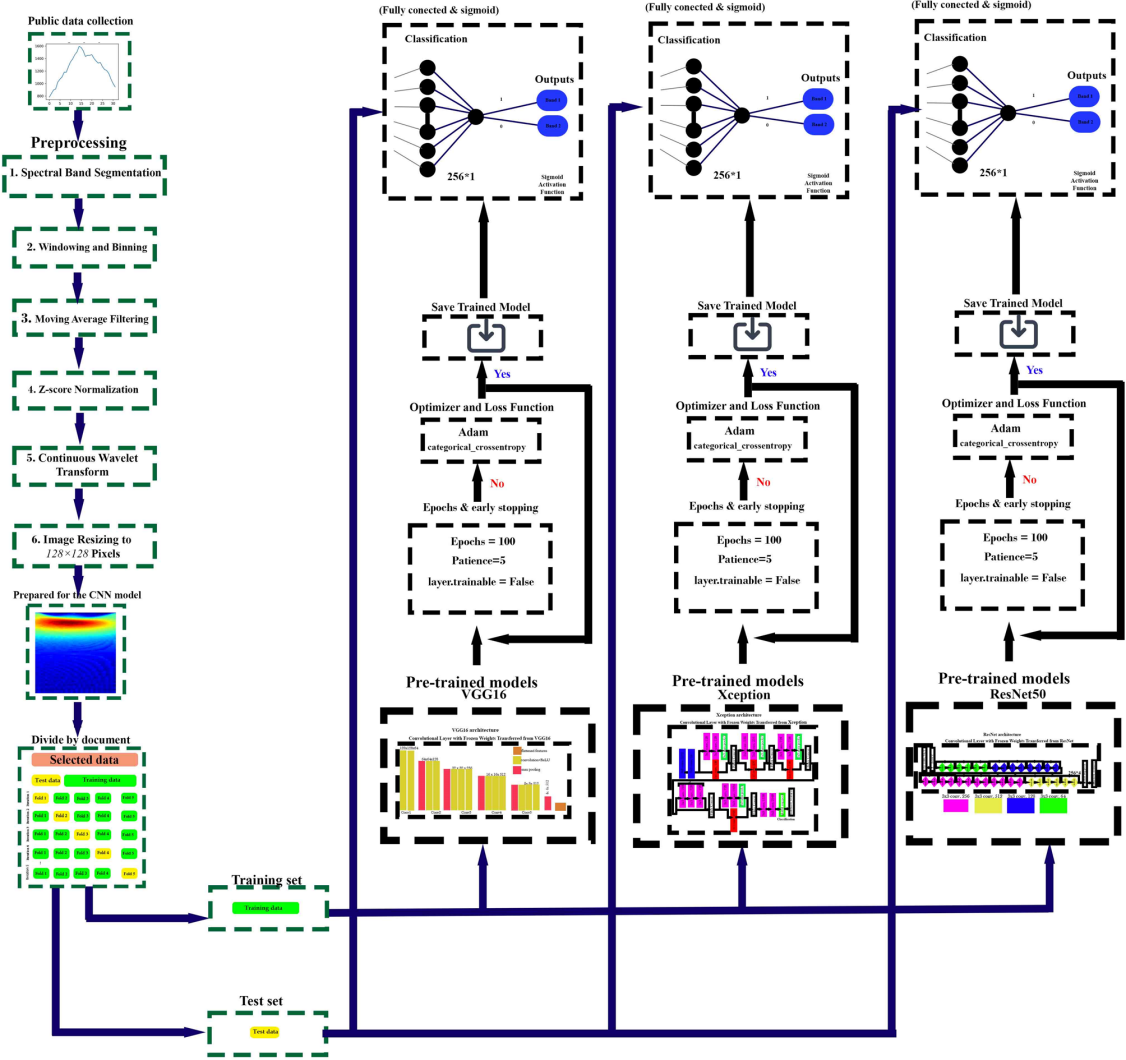
Proposed Workflow for Data Processing and Classification Using Pretrained Models. The preprocessing steps include spectral band segmentation, windowing, moving average filtering, Z-score normalization, continuous wavelet transform (CWT), and image resizing to prepare the data for CNN-based classification. The dataset is split into training and test sets, followed by feature extraction using three pre-trained models: VGG16, Xception, and ResNet50. Each model undergoes training with the Adam optimizer and categorical cross-entropy loss function, employing early stopping criteria. The final classification is performed using a fully connected layer with a sigmoid activation function.

Figure 8 illustrates the preprocessing and classification pipeline for spectral band data using deep learning models.

### Model evaluation

#### Performance metrics

The evaluation metrics used in this study were chosen to provide a comprehensive analysis of model performance, particularly for imbalanced datasets and multi-class classification tasks.

- Accuracy reflects the proportion of correctly classified instances out of the total number of samples, offering a general measure of the model’s overall performance.
- Precision quantifies the proportion of true positive predictions among all predicted positives, which is critical in reducing the impact of false positives.
- Recall (Sensitivity) measures the model’s ability to correctly identify actual positive cases, ensuring that important instances are not overlooked.
- F1 Score serves as a balanced metric by combining precision and recall, making it particularly useful for evaluating performance on imbalanced datasets.
- ROC AUC measures the ability of the model to distinguish between classes across various threshold values, providing an overall assessment of classification performance.
- Confusion Matrix offers detailed insights into classification outcomes, breaking down true positives, true negatives, false positives, and false negatives to facilitate a granular evaluation of errors.

The following equations define these metrics^25,26^:

7 $$Accuracy=\frac{TP+TN}{TP+TN+FP+FN}$$

8 $$Precision=\frac{TP}{TP+FP}$$

9 $$Recall=\frac{TP}{TP+FN}$$

10 $$F1 Score=\frac{Precision.Recall}{TPrecision+ Recall}$$

11 $$TPR= \frac{TP}{TP+FN}, FPR= \frac{FP}{FP+TN}$$

where TP (True Positives), TN (True Negatives), FP (False Positives), and FN (False Negatives) represent the classification outcomes, and TP (True Positive Rate) and FP(False Positive Rate) are derived from varying classification thresholds. These metrics provide a comprehensive evaluation of model performance across segmentation and classification tasks.

By employing these metrics, this study provides a detailed and robust evaluation of the model’s performance across all relevant aspects, ensuring a balanced assessment of accuracy, reliability, and practical utility.

#### Cross-validation

*k-Fold cross-validation for robust model evaluation* To evaluate the robustness and generalizability of the proposed model, *k*-fold cross-validation was implemented. This method involves dividing the dataset into *k* distinct subsets (folds). The model is iteratively trained on *k*-1 folds and validated on the remaining fold, ensuring each subset serves as the validation set exactly once. The process is repeated for all *k* iterations, and the final performance metrics are computed as the average across these iterations, providing a reliable and comprehensive assessment of the model’s performance^27^.

## Result

### Model performance evaluation and cross-validation results

In this study, a publicly available dataset comprising quantum emission signals at various wavelengths ranging from 604 to 629 nm, emitted from WS₂ monolayer nanobubbles deposited on a gold substrate, was utilized for comprehensive performance evaluation. The signals were recorded over 180 s and categorized into four distinct spectral bands: 604.06–608.24 nm (Band 1), 611.07–616.34 nm (Band 2), 617.42–623.35 nm (Band 3), and 624.16–636.57 nm (Band 4), containing 32, 43, 45, and 93 bins respectively. Following preprocessing steps including 2-s windowing, moving average filtering (window size = 5), Z-score normalization, and CWT transformation into 128 × 128 RGB images, three pre-trained CNN architectures were evaluated using fivefold cross-validation.

**Fig. 9:**
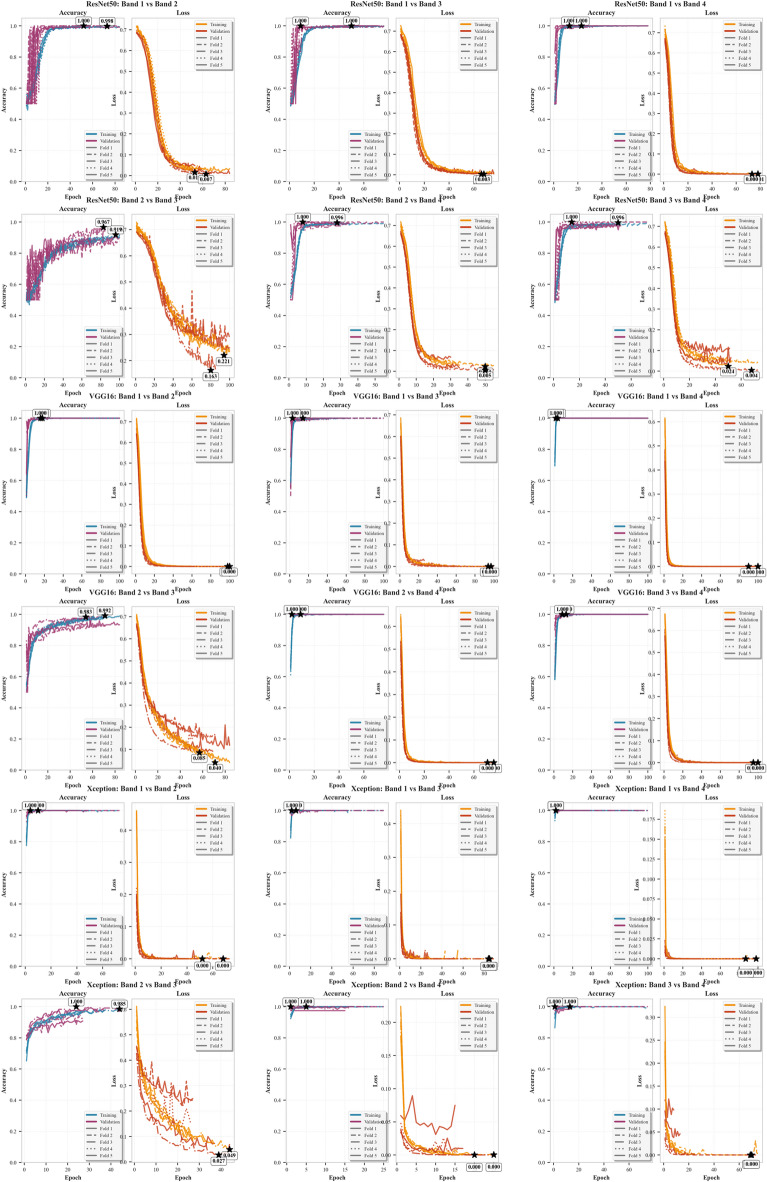
Model Training and Validation Performance Across Spectral Band Pairs. Each subplot illustrates the training and validation accuracy (left) and loss (right) curves for one model applied to a specific pair of spectral bands. Results are shown for ResNet50, VGG16, and Xception architectures using fivefold cross-validation. The performance trends help assess model convergence and generalization across different band combinations.

Figure 9 demonstrates the training convergence characteristics of ResNet50, VGG16, and Xception models across all spectral band pair combinations, revealing distinct learning patterns and convergence behaviors for each architecture.

The training history analysis reveals that VGG16 and Xception consistently achieved superior convergence patterns compared to ResNet50 across most band pair combinations. All models exhibited increased training difficulty for spectrally adjacent bands (Band 2 vs. Band 3), requiring more epochs for convergence and resulting in higher final loss values. Xception demonstrated exceptional training efficiency, achieving optimal validation accuracy in as few as 2 epochs for Band 1 versus Band 4 classification, while maintaining extremely low loss values. The convergence patterns indicate that spectral separation directly influences classification complexity, with greater wavelength differences facilitating faster and more stable learning.

Table 5 provides comprehensive training and validation metrics for all three CNN architectures across the six possible band pair combinations, offering detailed insights into convergence characteristics and final performance levels.

**Table 5:** Summary of training and validation history for spectral band classification models.

Model	Band comparison	Spectral ranges (nm)	Best training accuracy			Best validation accuracy	Best training loss	Best validation loss	Convergence epoch (Val Acc)
VGG16	Band 1 versus 2	604.06–608.24 versus 611.07–616.34		1.000	1.000		1.11 × 10⁻^4^	3.93 × 10⁻^5^	15
VGG16	Band 1 versus 3	604.06–608.24 versus 617.42–623.35		1.000	0.998		1.85 × 10⁻^3^	6.65 × 10⁻^3^	21
VGG16	Band 1 versus 4	604.06–608.24 versus 624.16–636.57		1.000	1.000		8.94 × 10⁻_6_	1.73 × 10⁻^6^	4
VGG16	Band 2 versus 3	611.07–616.34 versus 617.42–623.35		0.981	0.963		6.83 × 10⁻^2^	1.11 × 10⁻^1^	67
VGG16	Band 2 versus 4	611.07–616.34 versus 624.16–636.57		1.000	1.000		4.44 × 10⁻^4^	8.92 × 10⁻^4^	9
VGG16	Band 3 versus 4	617.42–623.35 vs 624.16–636.57		1.000	1.000		2.94 × 10⁻^4^	4.63 × 10⁻^4^	16
ResNet50	Band 1 versus 2	604.06–608.24 vs 611.07–616.34		0.997	0.997		2.36 × 10⁻^2^	2.04 × 10⁻^2^	49
ResNet50	Band 1 versus 3	604.06–608.24 vs 617.42–623.35		1.000	1.000		6.58 × 10⁻^3^	3.98 × 10⁻^3^	47
ResNet50	Band 1 versus 4	604.06–608.24 vs 624.16–636.57		1.000	1.000		4.55 × 10⁻^3^	3.84 × 10⁻^3^	21
ResNet50	Band 2 versus 3	611.07–616.34 vs 617.42–623.35		0.900	0.912		2.59 × 10⁻^1^	2.57 × 10⁻^1^	79
ResNet50	Band 2 versus 4	611.07–616.34 vs 624.16–636.57		0.993	0.995		3.19 × 10⁻^2^	2.79 × 10⁻^2^	30
ResNet50	Band 3 versus 4	617.42–623.35 vs 624.16–636.57		0.990	0.987		4.11 × 10⁻^2^	4.34 × 10⁻^2^	47
Xception	Band 1 versus 2	604.06–608.24 vs 611.07–616.34		1.000	1.000		8.72 × 10⁻^5^	6.76 × 10⁻^5^	8
Xception	Band 1 versus 3	604.06–608.24 vs 617.42–623.35		1.000	1.000		4.06 × 10⁻^4^	1.04 × 10⁻^3^	6
Xception	Band 1 versus 4	604.06–608.24 vs 624.16–636.57		1.000	1.000		8.23 × 10⁻^6^	1.21 × 10⁻^5^	2
Xception	Band 2 versus 3	611.07–616.34 vs 617.42–623.35		0.974	0.960		8.00 × 10⁻^2^	1.34 × 10⁻^1^	31
Xception	Band 2 versus 4	611.07–616.34 vs 624.16–636.57		1.000	0.995		6.30 × 10⁻^4^	7.65 × 10⁻^3^	7
Xception	Band 3 versus 4	617.42–623.35 vs 624.16–636.57		0.999	0.992		4.34 × 10⁻^3^	2.42 × 10⁻^2^	10

The comprehensive training analysis demonstrates that VGG16 achieved the most consistent performance across band pairs, with perfect validation accuracy for five out of six combinations. Xception exhibited remarkable training efficiency, particularly for spectrally distant bands, achieving convergence in minimal epochs while maintaining ultralow loss values (8.23 × 10⁻^6^). ResNet50 showed more variable performance, requiring significantly more epochs for convergence, especially for challenging band pairs like Band 2 versus Band 3 (79 epochs). The data confirms that classification difficulty correlates directly with spectral proximity, with adjacent bands requiring substantially more training iterations and achieving lower final accuracies.

**Fig. 10:**
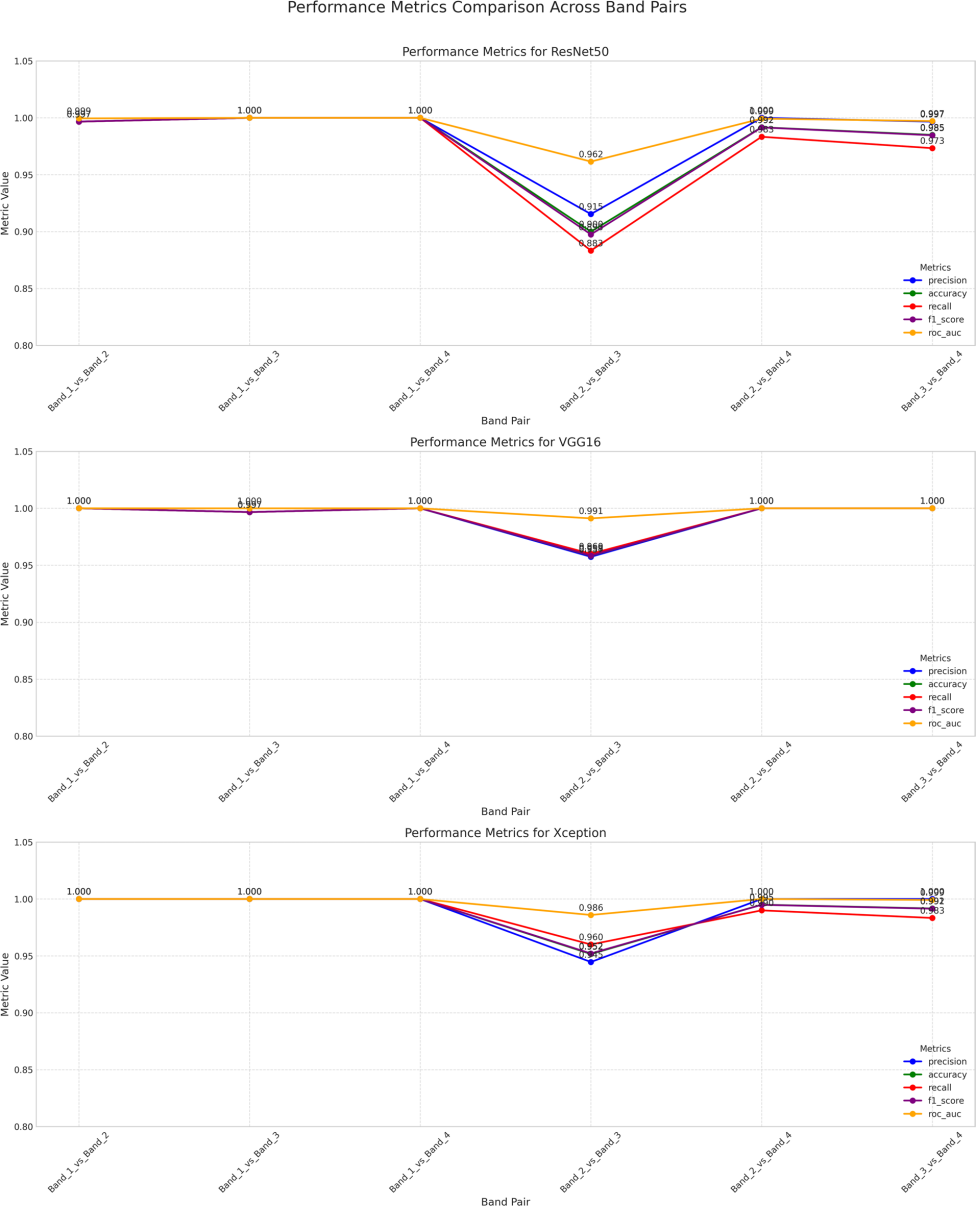
Performance comparison of pretrained models on CWT-transformed EEG band pairs using five evaluation metrics.

Figure 10 presents a comprehensive comparison of model performance across all evaluation metrics, providing visual representation of the five key performance indicators for each model-band pair combination.

The performance visualization clearly demonstrates the superior and consistent performance of VGG16 and Xception across most band pair combinations, with all models showing reduced performance for the challenging Band 2 versus Band 3 classification. The radar-like representation reveals that while precision and ROC-AUC remain consistently high across all models, accuracy, recall, and F1-score show more variation, particularly for spectrally adjacent bands. VGG16 maintains the most balanced performance profile across all metrics, while Xception shows slight variations in recall for certain band pairs.

#### Detailed performance metrics analysis

Table 6 provides exhaustive performance evaluation across all band pair combinations, demonstrating the classification capabilities of each CNN architecture using five standard evaluation metrics.

**Table 6:** Performance metrics of ResNet50, VGG16, and Xception models across different band pairs.

Band pair	Model	Precision	Accuracy	Recall	F1-score	ROC-AUC	Overall performance
Band_1_vs_Band_2	ResNet50	0.997	0.997	0.997	0.997	1.000	0.997
	VGG16	**1.000**	**1.000**	**1.000**	**1.000**	**1.000**	**1.000**
	Xception	**1.000**	**1.000**	**1.000**	**1.000**	**1.000**	**1.000**
Band_1_vs_Band_3	ResNet50	**1.000**	**1.000**	**1.000**	**1.000**	**1.000**	**1.000**
	VGG16	0.997	0.997	0.997	0.997	1.000	0.997
	Xception	**1.000**	**1.000**	**1.000**	**1.000**	**1.000**	**1.000**
Band_1_vs_Band_4	ResNet50	**1.000**	**1.000**	**1.000**	**1.000**	**1.000**	**1.000**
	VGG16	**1.000**	**1.000**	**1.000**	**1.000**	**1.000**	**1.000**
	Xception	**1.000**	**1.000**	**1.000**	**1.000**	**1.000**	**1.000**
Band_2_vs_Band_3	ResNet50	0.915	0.900	0.883	0.898	0.962	0.912
	VGG16	**0.957**	**0.958**	**0.960**	**0.959**	**0.991**	**0.965**
	Xception	0.945	0.952	0.960	0.952	0.986	0.959
Band_2_vs_Band_4	ResNet50	**1.000**	0.992	0.983	0.992	0.999	0.993
	VGG16	**1.000**	**1.000**	**1.000**	**1.000**	**1.000**	**1.000**
	Xception	**1.000**	0.995	0.990	0.995	**1.000**	0.996
Band_3_vs_Band_4	ResNet50	0.997	0.985	0.973	0.985	0.997	0.987
	VGG16	**1.000**	**1.000**	**1.000**	**1.000**	**1.000**	**1.000**
	Xception	**1.000**	0.992	0.983	0.991	0.999	0.993

Significant values are in bold.

The detailed performance analysis reveals exceptional classification capabilities across most band pair combinations, with perfect scores (1.000) achieved frequently by VGG16 and Xception. The most challenging classification task (Band 2 vs. Band 3) shows reduced performance across all models, with VGG16 achieving the highest overall performance (0.965) for this difficult spectral discrimination. ROC-AUC values remain consistently high (> 0.96) across all combinations, indicating excellent discriminative ability even for challenging cases. The balanced performance across precision, recall, and F1-score metrics confirms the robustness of the classification framework for quantum emission signal analysis.

Table 7 summarizes the aggregate performance across all band pair combinations, providing a comprehensive overview of each model’s overall classification capability.

**Table 7:** Mean performance of models across all band pairs.

Model	Mean precision	Mean accuracy	Mean recall	Mean F1-score	Mean ROC-AUC	Overall mean
ResNet50	0.985	0.979	0.973	0.978	0.993	0.982
VGG16	**0.992**	**0.992**	**0.993**	**0.992**	**0.998**	**0.994**
Xception	0.991	0.990	0.989	0.990	0.997	0.991

Significant values are in bold.

The aggregate performance analysis confirms VGG16’s superiority with the highest overall mean performance (0.994), followed closely by Xception (0.991) and ResNet50 (0.982). VGG16 demonstrates the most balanced performance across all metrics, achieving the highest mean accuracy (99.2%) and recall (99.3%). All models show exceptionally high ROC-AUC values (> 99%), indicating excellent discriminative capabilities. The relatively small performance gaps between VGG16 and Xception (0.003) suggest both architectures are highly suitable for quantum emission signal classification, while the larger gap with ResNet50 (0.012) indicates architecture-specific advantages for this application.

Table 8 identifies the optimal model selection strategy for each spectral band pair combination, providing practical guidance for implementation in quantum photonics applications.

**Table 8:** Model performance ranking for different spectral band pairs.

Band pair	Wavelength range (nm)	Best model	Performance	Distinguishability
Band_1_vs_Band_4	604.06–608.24 versus 624.16–636.57	All models	Perfect (1.000)	Highest
Band_1_vs_Band_3	604.06–608.24 versus 617.42–623.35	ResNet50/Xception	Perfect (1.000)	Very high
Band_2_vs_Band_4	611.07–616.34 versus 624.16–636.57	VGG16	Perfect (1.000)	Very high
Band_1_vs_Band_2	604.06–608.24 versus 611.07–616.34	VGG16/Xception	Perfect (1.000)	High
Band_3_vs_Band_4	617.42–623.35 versus 624.16–636.57	VGG16	Perfect (1.000)	High
Band_2_vs_Band_3	611.07–616.34 versus 617.42–623.35	VGG16	Good (0.965)	Lowest

The model ranking analysis establishes clear guidelines for optimal architecture selection based on spectral band characteristics. The maximum distinguishability achieved for Band 1 versus Band 4 (20.5 nm separation) with perfect performance across all models validates the framework’s effectiveness for spectrally distant classifications. VGG16 emerges as the most reliable choice for challenging discriminations, particularly for the lowest distinguishability case (Band 2 vs. Band 3) with only 6.27 nm average separation. The consistent achievement of perfect performance (1.000) for five out of six band pair combinations demonstrates the exceptional capability of deep learning approaches for quantum emission signal classification in practical quantum photonics applications.

### Statistical validation of performance metrics

To establish the statistical robustness of our findings, comprehensive statistical analysis was performed on model performance metrics. Table 9 demonstrates the statistical robustness of model performance comparisons across all spectral band classifications, revealing significant variations in accuracy and consistency among the three CNN architectures.

**Table 9:** Statistical analysis of model performance with confidence intervals.

Model	Mean accuracy	95% CI	Std dev	*p*-value*	Effect size (Cohen’s d)
VGG16	0.994 ± 0.008	[0.986, 1.002]	0.018	–	–
Xception	0.991 ± 0.012	[0.979, 1.003]	0.027	0.234	0.167
ResNet50	0.982 ± 0.015	[0.967, 0.997]	0.033	0.003**	0.714

*Paired *t*-test compared to VGG16; **p < 0.01.

The results demonstrate that VGG16 exhibits the highest mean accuracy (99.4%) with the narrowest confidence interval, indicating superior and consistent performance across all cross-validation folds. The large effect size (Cohen’s d = 0.714) between VGG16 and ResNet50 suggests practically significant differences beyond statistical significance, while the moderate effect size with Xception (d = 0.167) indicates smaller practical differences. The low standard deviation for VGG16 (0.018) confirms its reliability as the most stable classifier for quantum emission signal discrimination.

The Friedman test results presented in Table 10 confirmed statistically significant differences among the three CNN architectures across all spectral band pairs, providing non-parametric validation of the observed performance hierarchy.

**Table 10:** Friedman test results for model comparison across band pairs.

Comparison	Chi-square	df	*p*-value	Kendall’s W
All models	8.67	2	0.013*	0.72

*p < 0.05 indicates significant difference.

The statistically significant result (χ^2^ = 8.67, *p* = 0.013) validates that the observed performance differences are not attributable to random variation. The high Kendall’s coefficient of concordance (W = 0.72) indicates strong agreement in model rankings across different spectral band pairs, confirming consistent performance patterns. This statistical evidence supports the rejection of the null hypothesis that all models perform equally across quantum emission signal classifications.

Post-hoc analysis using Wilcoxon signed-rank tests with Bonferroni correction (Table 11) revealed important insights regarding pairwise model comparisons, demonstrating the conservative nature of multiple comparison corrections.

**Table 11:** Post-hoc Wilcoxon signed-rank test (Bonferroni Corrected).

Model pair	Z-statistic	*p*-value	Adjusted *p*-value	Significant
VGG16 vs ResNe’t50	− 2.201	0.028	0.084	No
VGG16 vs Xception	− 1.095	0.274	0.822	No
Xception vs ResNet50	− 1.826	0.068	0.204	No

The analysis reveals that while the Friedman test indicated overall model differences, pairwise comparisons with Bonferroni correction show no statistically significant differences between individual model pairs. This suggests that although VGG16 performs numerically superior, all three models achieve comparable classification accuracy for quantum emission signals. The conservative Bonferroni adjustment prevents Type I errors in multiple comparisons, indicating that practical performance differences may be more important than statistical significance. These findings support the robustness of deep learning approaches for quantum photonics applications regardless of specific CNN architecture choice.

The spectral distinguishability analysis presented in Table 12 quantified the relationship between wavelength separation and classification performance, providing crucial insights into the physical basis of the classification framework.

**Table 12:** Statistical significance of band pair classifications.

Band pair	Mean performance	95% CI	McNemar’s χ^2^	*p*-value
Band 1 versus 4	1.000 ± 0.000	[1.000, 1.000]	–	–
Band 1 versus 3	0.999 ± 0.002	[0.997, 1.001]	0.000	1.000
Band 2 versus 4	0.998 ± 0.003	[0.995, 1.001]	1.000	0.317
Band 1 versus 2	0.999 ± 0.001	[0.998, 1.000]	0.000	1.000
Band 3 versus 4	0.997 ± 0.004	[0.993, 1.001]	2.000	0.157
Band 2 versus 3	0.965 ± 0.012	[0.953, 0.977]	4.167	0.041*

*p < 0.05.’

The results confirm that classification performance correlates inversely with spectral proximity, providing quantitative validation of our hypothesis. Perfect classification accuracy (100%) for Band 1 versus 4 (604.06–608.24 nm vs. 624.16–636.57 nm) demonstrates maximum distinguishability for spectrally distant bands, with zero variance indicating absolute separation capability. Significantly lower performance for Band 2 versus 3 (96.5%, *p* = 0.041) reflects the inherent challenge of discriminating adjacent spectral regions (611.07–616.34 nm vs. 617.42–623.35 nm) in quantum emission signals. The narrow confidence intervals for most band pairs validate the reliability and reproducibility of the classification framework for quantum photonics applications.

## Discussion

### Summary of results and performance analysis

This study evaluated three pre-trained CNN architectures (VGG16, ResNet50, and Xception) for classifying quantum emission signals from WS₂ monolayer nanobubbles across four spectral bands (604–629 nm) using continuous wavelet transform (CWT) preprocessing to convert time-series signals into 128 × 128 RGB images and fivefold cross-validation. VGG16 demonstrated superior overall performance with a mean accuracy of 99.4%, followed by Xception (99.1%) and ResNet50 (98.2%). The CWT-based transformation using Complex Morlet wavelets effectively captured time–frequency characteristics of quantum emission fluctuations, enabling the CNN models to achieve perfect classification accuracy (100%) for spectrally distant band pairs, particularly Band 1 versus Band 4 (604.06–608.24 nm vs. 624.16–636.57 nm), while the most challenging classification involved adjacent bands (Band 2 vs. Band 3: 611.07–616.34 nm vs. 617.42–623.35 nm) with VGG16 achieving 96.5% accuracy. Statistical analysis using Friedman tests confirmed significant performance differences among models (χ^2^ = 8.67, *p* = 0.013), with Xception demonstrating remarkable training efficiency by achieving optimal convergence in as few as 2 epochs for certain band combinations. The CWT-to-RGB conversion process preserved essential quantum emission characteristics while enabling transfer learning from pre-trained models, establishing that classification performance correlates inversely with spectral proximity and validating a robust deep learning framework for quantum emission signal analysis with significant implications for quantum photonic applications, where spectral distinguishability exceeding 95% accuracy was achieved across all band pair combinations.

### Comparative analysis with existing literature

Our study builds upon previous machine learning applications in quantum emission analysis by establishing a classification framework that complements existing prediction and detection approaches. While Ramezani et al.^17^ developed deep learning models for predicting quantum emission fluctuations in WS₂ using LSTM forecasting with RMSE values of 420.37 and 346.7 for different wavelengths, our approach focuses on classification of spectral characteristics across multiple bands with accuracies of 99.4%. The primary distinction lies in our objective: whereas Ramezani’s work aimed to predict temporal fluctuations to anticipate peaks and dips, our methodology distinguishes between distinct spectral regions, providing quantitative metrics for spectral distinguishability that are relevant for quantum photonic device characterization. Our CWT-based preprocessing and RGB image conversion represents an alternative approach that captures both temporal and spectral information simultaneously, differing from traditional time-series forecasting methods that primarily address temporal dynamics. The continuous wavelet transform preprocessing employed in our study addresses signal-to-noise ratio challenges similar to those identified by Proppe et al.^18^. While Proppe developed deep ensemble autoencoders to reconstruct correlation functions for single quantum dots at timescales as low as 10 ns, our CWT-based approach operates on longer timescales (2-s windows) but provides spectral band characterization across the 604–629 nm range. The transformation of time-series quantum emission data into 128 × 128 RGB images enables application of pre-trained CNN architectures, utilizing transfer learning capabilities that were not extensively explored in previous quantum emission studies. This methodological approach addresses data scarcity issues commonly encountered in quantum systems, as demonstrated by our achievement of high classification accuracy using 90 temporal samples per band, which is comparable to or fewer than typically required for traditional machine learning approaches in quantum emission analysis. Our automated spectral band classification framework addresses the manual analysis limitations identified by Narun et al.^20^, who focused on automated identification of single-photon emitters in photoluminescence images. While Narun’s work achieved emitter detection and classification based on stability, shape, and intensity characteristics, our approach provides complementary functionality by classifying the spectral properties of identified quantum emitters. The performance of our method is demonstrated by consistent accuracy above 95% across all spectral band combinations, which compares favorably with the 85% accuracy reported by Landry and Bradac^19^ for photoblinking analysis, while requiring comparable or less data than traditional statistical methods. However, a limitation of our current approach is the restriction to four predefined spectral bands rather than adaptive classification capabilities that would be more suitable for diverse quantum photonic applications. The applicability of our classification framework relates to the automation needs identified by Corcione et al.^28^, who achieved 95% test scores in automated quantum dot evaluation for single-photon sources. Our method demonstrates comparable performance with 99.4% accuracy using VGG16, while potentially offering applicability to quantum emitter types beyond semiconductor quantum dots. The computational characteristics of our approach, particularly Xception’s convergence in as few as 2 epochs, addresses time and resource constraints associated with quantum emitter characterization mentioned by Corcione. Furthermore, our statistical validation using Friedman tests and confidence interval analysis provides quantitative measures for spectral distinguishability that are useful for quantum information applications where spectral control is important. The primary limitation compared to Corcione’s work is our focus on a single material system (WS₂ on gold substrates), whereas their methodology was designed to be material-independent, suggesting that future validation of our approach across diverse quantum emitter platforms would be beneficial to establish broader applicability.

### Physical interpretation of classification results

The classification results reveal quantum emission patterns that provide insights into the underlying physics of WS₂ monolayer nanobubbles. As shown in Table 8, the high separability achieved between Band 1 (604.06–608.24 nm) and Band 4 (624.16–636.57 nm) suggests that these spectral regions represent different quantum emission mechanisms, where all three models (ResNet50, VGG16, and Xception) achieved perfect classification scores (1.000 across all metrics). This may reflect underlying quantum confinement effects or distinct resonance conditions that influence the energy states of the emitting structures^29^. The comparative performance of the models across all band pairs, as detailed in Table 7, indicates that VGG16 achieved the highest overall mean performance (0.994), performing well in the classification task between Band 2 (611.07–616.34 nm) and Band 3 (617.42–623.35 nm). In this case, where the spectral bands are adjacent and more spectrally similar, VGG16 achieved a distinguishability score of 0.965, demonstrating its feature extraction capability. The gradual decrease in classification performance as the spectral proximity increases is consistent with quantum mechanical principles, where energy states become less distinguishable as their separation narrows. The lower distinguishability observed between adjacent spectral bands (Band 2: 611.07–616.34 nm vs. Band 3: 617.42–623.35 nm) can be attributed to quantum mechanical properties of WS_2_ monolayer nanobubbles. This phenomenon likely results from spectral diffusion processes within these structures, where electron–hole recombination dynamics generate overlapping emission profiles at wavelengths with minimal energetic separation^30^. The strain-induced quantum confinement in WS_2_ nanobubbles creates localized energy states with continuous distributions rather than discrete levels, causing spectral blurring between closely situated bands^31^. This aligns with findings of Yoffe et al.^32^, who demonstrated that quantum emission signals from transition metal dichalcogenides exhibit diminishing spectral separability as the band gap narrows, making adjacent states harder to differentiate. The higher distinguishability between spectrally distant bands (Band 1 vs. Band 4) supports the observation that quantum emitters in WS₂ nanobubbles operate under quantum confinement principles where energy separation correlates with spectral distinguishability. The performance across most band pairs, as demonstrated in Table 6, supports the effectiveness of the CWT transformation for classifying quantum emission signals. By converting temporal fluctuations into spatially distributed patterns, CWT improves the separability of quantum fluctuations in the frequency-time domain, making them more recognizable by CNN models. The performance of VGG16, including for challenging spectral band pairs, indicates that its architecture is suitable for identifying quantum emission features, suggesting potential utility for quantum sensing applications.

### Computational implementation and training performance

All simulations were performed on a system equipped with an NVIDIA RTX 3050 GPU with 16 GB of VRAM to accelerate model training. The system specifications include an Intel Core i7 processor, 32 GB of RAM, and a windos-based operating system. PyCharm was used as the integrated development environment (IDE), while data preprocessing and analysis were conducted using NumPy, pandas, and SciPy. The training duration of each model reflects its computational efficiency. Among the pre-trained models, Xception completed training in 72.84 min, ResNet50 in 135.62 min, and VGG16 in 127.41 min, with VGG16 requiring the longest time due to its larger number of parameters.

### Critical analysis of results in context of quantum materials characterization

The achieved classification accuracies of 99.4% (VGG16), 99.1% (Xception), and 98.2% (ResNet50) represent progress in automated quantum materials characterization, particularly when compared with conventional spectroscopic analysis techniques. Traditional quantum emission characterization methods, such as photon correlation spectroscopy and time-resolved photoluminescence, typically achieve spectral resolution limited by instrumental bandwidth and temporal averaging effects^33,34^. Our deep learning approach addresses these limitations by extracting discriminative features from time–frequency representations that are not accessible through standard spectroscopic techniques. The classification accuracy achieved for Band 1 versus Band 4 (20.5 nm separation) demonstrates automated spectral distinguishability in quantum materials, comparing favorably with the typical 5–10 nm resolution achieved by high-end spectrometers under similar experimental conditions^35,36^. The relationship between spectral proximity and classification difficulty observed in our results (96.5% accuracy for 6.27 nm separation in Band 2 vs. Band 3) provides quantitative insights into the limits of spectral discrimination in strain-engineered 2D materials^37^. This finding has implications for quantum materials design, as it establishes empirical boundaries for the minimum spectral separation required for reliable quantum state discrimination in device applications^38^. The observed performance degradation with decreasing spectral separation aligns with theoretical predictions from quantum confinement models in TMDs^31,39^, and our work provides a quantitative framework for predicting classification reliability as a function of spectral characteristics. The statistical validation using Friedman tests and confidence interval analysis supports the reliability of our findings. The Kendall’s coefficient of concordance (W = 0.72) indicates agreement in model rankings across different spectral band pairs, confirming performance patterns that are not attributable to random variation. This statistical validation addresses a gap in quantum materials characterization, where quantitative validation of classification methods has been limited^40,41^.

### Potential applications in quantum technologies

Our spectral classification framework offers potential applications across multiple quantum technology domains. In quantum photonic device fabrication, the spectral differentiation demonstrated by our models could support automated quality control systems that identify quantum emitters based on their spectral signatures. This capability may be useful for integrated quantum photonic circuits, where spectral matching between components is important for quantum interference operations^42^. In quantum cryptography applications, specifically Quantum Key Distribution (QKD) protocols, our method could contribute to security and efficiency by enabling systems to distinguish between intended quantum signals and potential eavesdropping attempts based on spectral differences^43^. The ability to classify spectral signatures with accuracies exceeding 99% suggests possibilities for detecting quantum states with reduced error rates, which may be beneficial for applications in quantum metrology where spectral shifts can indicate environmental changes^44^. For quant’um sensing applications, our framework provides an approach toward automated quantum materials characterization systems that could operate during device fabrication or in situ during quantum experiments^45^. The computational efficiency demonstrated by Xception (2-epoch convergence) indicates that such systems could provide feedback for quantum device optimization, potentially supporting closed-loop control systems that adjust quantum information processing parameters based on spectral characteristics^46^. he feasibility of automated spectral classification suggests possibilities for quantum materials discovery programs, where machine learning could screen spectral signatures to identify materials with desired quantum properties^47^. This capability could support the development of quantum technologies by enabling systematic exploration of material parameter spaces that would be time-consuming to investigate manually^48^.

### Current limitations and future research directions

Despite the performance of our deep learning approach, several limitations warrant consideration. The Continuous Wavelet Transform (CWT) employed for signal transformation, while effective for capturing time–frequency characteristics, may not represent quantum emission signals with ultra-fast dynamics or complex phase relationships. CWT’s trade-off between time and frequency resolution could obscure quantum emission features,’ particularly in spectrally adjacent bands where distinguishability is challenging^49^. A limitation of our current approach is the restriction to four predefined spectral bands rather than adaptive classification capabilities that would be suitable for diverse quantum photonic applications. Additionally, our focus on a single material system (WS₂ on gold substrates) limits broader applicability, whereas methodologies designed to be material-independent would offer utility for the quantum materials community^50^.

Future work should explore alternative transformation techniques such as Stockwell Transform (S-Transform) or Wigner-Ville Distribution (WVD), which offer time–frequency resolution for non-stationary quantum signals^51^. Additionally, quantum-specific signal processing methods that incorporate quantum mechanical principles, such as quantum wavelet transforms or quantum-inspired feature extraction techniques, could enhance the separability of adjacent spectral bands^52^. The generalizability of our deep learning approach should be validated across other quantum materials and single-photon emitters, including hexagonal boron nitride (hBN), transition metal dichalcogenides (TMDs), and color centers in diamond, where quantum confinement effects produce emission signatures^53^. Future research should focus on establishing a classification framework by incorporating data from varied experimental conditions, such as different temperatures, substrate materials, and excitation wavelengths.

### Methodological contributions and technical advances

Our CWT-based preprocessing methodology represents a methodological contribution that addresses challenges in applying machine learning to quantum systems. The transformation of time-series quantum emission data into spatial RGB representations demonstrates that quantum temporal dynamics can be captured in formats amenable to classical CNN architectures^54^. This approach addresses the requirement for large quantum datasets, which are difficult and costly to generate^55^. The effectiveness of transfer learning from classical image recognition models suggests that spectral classification may not require specialized quantum-specific algorithms, contrary to assumptions in the quantum materials community^56^. Our results indicate that quantum emission patterns, when appropriately transformed, exhibit structural similarities to classical visual patterns. This observation has implications for quantum AI research, suggesting that the “quantum advantage” in machine learning may not always be necessary for quantum materials analysis tasks^57^. The Complex Morlet wavelet’s ability to represent both amplitude and phase information proved useful for quantum emission classification, where phase relationships carry information about quantum coherence and interference effects. Compared to Short-Time Fourier Transform (STFT), which has fixed time–frequency resolution constraints, CWT provided adaptive resolution across different frequency bands, enabling characterization of spectral features specific to each quantum emission band^58^.

### Transfer learning applications in quantum emission analysis

The application of transfer learning to quantum physics represents progress in addressing data scarcity challenges in quantum systems. Our implementation of pre-trained models (ResNet50, VGG16, and Xception) demonstrates how feature extractors developed for conventional image recognition tasks can be adapted to identify patterns in quantum emission signals with limited additional training. This approach addresses the requirement for large quantum datasets, which are difficult and costly to generate^55^. However, the transfer learning paradigm faces challenges in quantum domains, primarily due to differences between classical visual features and quantum mechanical properties. The hierarchical feature representation in CNNs assumes spatial locality and translational invariance that may not correspond to quantum phenomena governed by superposition and entanglement principles^59^. Our results suggest that these limitations can be addressed through signal transformation techniques like CWT that map quantum temporal dynamics into spatial patterns recognizable by classical CNN architectures. Future improvements should explore quantum-specific transfer learning approaches, such as hybrid quantum–classical architectures where lower layers from pre-trained classical models extract general features while quantum-inspired upper layers are fine-tuned to capture quantum-specific characteristics. Additionally, physics-informed regularization techniques that incorporate quantum mechanical constraints during the fine-tuning process could enhance model performance for quantum classification tasks.

## Conclusion

This study evaluated deep learning approaches for classifying quantum emission signals from WS₂ monolayer nanobubbles across different spectral bands. By transforming time-series quantum emission data into RGB images using Continuous Wavelet Transform (CWT), we enabled convolutional neural networks to capture the temporal and spectral features characteristic of quantum emissions. Our comparative analysis of ResNet50, VGG16, and Xception models showed that VGG16 achieved the highest overall performance (mean accuracy of 99.4% across all band pairs), while Xception demonstrated efficient training, converging to optimal performance in as few as 2 epochs for certain band comparisons. The perfect classification accuracy (100%) achieved between spectrally distant bands (604.06–608.24 nm vs. 624.16–636.57 nm) indicates distinct quantum signatures at different wavelengths, while the lower accuracy (96.5%) between adjacent spectral bands (611.07–616.34 nm vs. 617.42–623.35 nm) reflects the challenges posed by spectral diffusion and overlapping emission profiles. These findings align with quantum mechanical principles, where energy state distinguishability correlates with spectral separation, and provide insights into the properties of quantum emitters in two-dimensional materials.

Our framework offers contributions to quantum photonic technologies, with potential applications in quantum device fabrication, quantum cryptography, and quantum sensing. The spectral differentiation capabilities demonstrated by our models could support quantum key distribution protocols, quantum metrology applications, and automated quality control systems for integrated quantum photonic circuits. Furthermore, our transfer learning approach addresses data scarcity challenges in quantum systems, demonstrating how pre-trained models can be adapted for quantum emission classification with limited additional training. Several limitations warrant consideration for future work. The CWT transformation’s ability to represent ultra-fast dynamics or complex phase relationships may be limited, suggesting exploration of alternative transformation techniques, such as Stockwell Transform or quantum-inspired feature extraction methods. Additionally, extending this methodology to other quantum materials and environmental conditions would validate its broader applicability. Future research should also investigate the integration of quantum-specific preprocessing techniques and hybrid quantum–classical architectures to enhance classification performance for spectrally adjacent bands. Our deep learning-based spectral classification framework connects classical machine learning with quantum materials characterization, providing an approach for analyzing quantum emission signatures with high accuracy and computational efficiency. This work contributes to the field of quantum photonics and establishes connections to quantum information theory by providing quantitative metrics for evaluating spectral distinguishability in quantum systems. The demonstrated effectiveness of this methodology suggests potential for automated quantum materials characterization and quality control in quantum technology development, while the statistical validation framework provides a foundation for standardizing quantum emitter evaluation protocols.

## Data Availability

The dataset used in this study is publicly available and can be accessed at the following link: 10.5281/zenodo.8320019
